# Comparing the effects of multimedia and face-to-face pain management education on pain intensity and pain catastrophizing among patients with chronic low back pain: A randomized clinical trial

**DOI:** 10.1371/journal.pone.0269785

**Published:** 2022-06-16

**Authors:** Maryam Shaygan, Azita Jaberi, Roghayyeh Firozian, Zahra Yazdani

**Affiliations:** 1 Community Based Psychiatric Care Research Center, Shiraz University of Medical Sciences, Shiraz, Iran; 2 Student Research Committee, Shiraz University of medical sciences, Shiraz, Iran; 3 Department of Nursing, School of Nursing and Midwifery, Shiraz University of Medical Sciences, Shiraz, Iran; Prince Sattam Bin Abdulaziz University, College of Applied Medical Sciences, SAUDI ARABIA

## Abstract

**Introduction:**

Previous studies into Low Back Pain (LBP) assessed the effects of physical interventions or face-to-face (FTF) education mostly in western cultures. The present study aimed to compare the effects of multimedia and FTF pain management education (PME) on pain intensity and pain catastrophizing among participants with chronic LBP.

**Methods:**

This double-blind randomized controlled clinical trial was conducted on ninety participants with chronic LBP randomly allocated to either multimedia, FTF, or control groups. Participants in the multimedia group received PME through watching seven educational CDs at home and their counterparts in the FTF group received the same educations in seven weekly FTF educational sessions. Pain intensity (using a numerical rating scale) and pain catastrophizing (using the Pain Catastrophizing Scale) were assessed before, immediately after, and one month after the study intervention. The effects of the interventions were assessed using the repeated-measures multivariate analysis of variance (MANOVA). Effect size and minimal detectable change (MDC) were reported for both variables. The regression model used in the present study was Generalized Estimating Equations (GEE).

**Findings:**

The findings of MANOVA showed the significant effects of time on pain intensity and pain catastrophizing (P<0.001). The Tukey’s test showed that before and immediately after the intervention, the mean scores of pain intensity and pain catastrophizing in the FTF and PME groups were significantly different from the control group (P<0.001 and P = 0.001, respectively). MDC did not show clinically significant changes in the mean score of pain intensity and GEE revealed significant difference among the groups.

**Conclusion:**

The findings suggested that multimedia PME is as effective as FTF education in reducing pain intensity and pain catastrophizing among participants with LBP. Future studies into the effects of education on LBP are recommended to consider longitudinal designs, a reliable cutoff score for pain catastrophizing, and participants’ physical ability.

**IRCT registration code:**

IRCT20180313039074N1.

## Introduction

Low back pain (LBP) is a common type of chronic pain [1] which affects 57.6 million people in the world [2]. LBP imposes a heavy burden on healthcare systems so that almost three fourth of all pain-related healthcare costs are spent on LBP management [3]. It not only causes physical and financial problems, but also negatively affects the mental status and the social activities of afflicted patients and their families [4].

We know that chronic pain has different biopsychosocial aspects and hence, its management should be based not only on physical factors, but also on psycho-social factors [5]. The Fear Avoidance Model illustrates that some psychological factors like pain catastrophizing can lead to avoidance, depression, and disability among patients with chronic pain [6].

Pain catastrophizing is a cognitive process of exaggerating or magnifying pain perception which results in greater attention to the negative aspects of pain, interpretation of physical arousals as pain symptoms, and pain intensity [7]. Besides pain catastrophizing, factors such as stress, anger, poor communication skills, and limited assertiveness can affect pain intensity among patients with chronic conditions. For example, a study found stress, social conflicts, and non-assertive relationships as aggravating factors of chronic pain [8]. Studies into occupational therapy for chronic pain also emphasize that patients with limited problem solving, communication, and assertiveness skills may have problems in pain management [9]. Consequently, besides physical interventions, education of psychological skills, mainly stress management, assertiveness, effective communication, positive thinking, and anger management, should be included in pain management programs [10].

Previous studies reported contradictory results respecting the effects of outpatient psychological interventions such as cognitive behavioral therapy on chronic pain management in adults [11]. A systematic review on nineteen studies found that there was no clear evidence concerning the effects of cognitive behavioral therapy, biofeedback, and relaxation on pain intensity and disability among patients with neck pain [12]. A recent meta-analysis on eleven clinical trials also found no significant difference between the long-term effects of psychological interventions and standard interventions on pain intensity [13]. Besides contradictory results, previous studies mostly focused on one aspect of pain (either physical or psychological), while chronic pain is a complex experience and hence multidisciplinary interventions are needed for its effective management [1]. On the other hand, patients who live far from pain management centers may have limited access to multidisciplinary pain management services. Therefore, tele-nursing methods including multimedia education are needed to improve patient access to pain management services and remove the barriers to evidence-based treatments.

Some previous studies evaluated the effects of technology-based pain management programs [14, 15]. However, most of them were web-based programs [14, 16], single-center, and non-controlled [12, 13]. Moreover, there are limited comparative studies into the effects of FTF and multimedia pain management programs [17, 18]. On the other hand, the results of the existing studies in this area may not be generalizable to different context because the effects of psychological factors on chronic pain largely depend on patients’ sociocultural background [19]. Nonetheless, most previous studies into pain catastrophizing and pain were conducted in northern European countries and Anglo-Saxon cultures [20, 21]. There is limited information in this area in the culture of Asian countries, particularly in Iran. Therefore, studies in different contexts and cultures are needed to provide more reliable results regarding the effects of multimedia pain management program on chronic LBP and pain catastrophizing [19]. All these gaps highlight the necessity of conducting further studies into pain catastrophizing among patients with chronic LBP and the comparative effects of face-to-face and multimedia treatments in this area. The present study was designed and conducted to reduce these gaps. The aim of the current study was to compare the effects of multimedia and face to face (FTF) pain management education (PME) on pain intensity and pain catastrophizing among participants with chronic LBP. The hypothesis of the present study was that participants with chronic back pain who receive FTF and multimedia PME will show significant decrease in pain intensity and pain catastrophizing, assessed immediately after program and at 1-month follow-up.

## Methods

### Study design

This randomized controlled trial was conducted using a parallel-group (1:1:1) design and in accordance with the CONSORT 2010 guideline [22].

### Participants and setting

The study was conducted from September 2019 to February 2020 in three healthcare centers affiliated to Fasa University of Medical Sciences, Fasa, Iran. Study participants were participants with non-specific chronic LBP. Inclusion criteria were age over eighteen years, agreement for participation, ability to use educational CDs, ability to attend the study setting for participation in FTF sessions, no history of other types of chronic pain (e.g. chronic headache), and chronic psychiatric disorders (such as schizophrenia), and definite diagnosis of non-specific chronic LBP established at least one month before the study by a medical specialist based on the data obtained through physical examination and diagnostic procedures such as simple radiography, computed tomography, or magnetic resonance imaging. Exclusion criteria were voluntary withdrawal from the study for any reason (e.g. severe LBP), participation in any other educational program on pain during the study, and more than two absences from the educational sessions of the FTF intervention.

### Sample size calculation

Sample size was calculated with a confidence level of 0.95, a power of 0.80, and a probable attrition rate of 10% and based on the findings of a former study regarding the mean score of pain catastrophizing in chronic LBP (M1 = 14.4, M2 = 11.78, S1 = 5.6, S2 = 3.9) [23]. Accordingly, the MedCalc software revealed that 36 participants per group were needed.

### Recruitment

Participants with chronic LBP were selected from three healthcare centers. Study staff (not involved in the group assignment) contacted (by telephone) the participants and asked them whether they were willing to participate in the study. The eligible participants were screened until the target number of 108 participants was reached (36 participants in each group). All participants were informed about the voluntary nature of their participation, and informed consent was obtained from them.

### Randomization

Participants were randomly allocated to either the multimedia, FTF, or the wait-list control group. A research assistant not involved in the current study performed random allocation through block randomization with a block size of 6 and cards labeled A, B, and C. Allocation concealment was ensured using sequentially numbered, opaque, sealed envelopes (SNOSE) [24].

### Blinding

The participants were blinded to the group assignments and did not know what the other interventions were. In addition, the evaluator and the analyzer of the outcomes were not informed about the treatment assignments.

### Study groups

#### Multimedia pain management education

Eligible participants in this group received multimedia PME in seven weeks. The modules of this education consisted of definition of chronic pain and the psychological factors affecting pain experience (the first session), appropriate physical exercises for chronic LBP (the second session), effective communication skills (the third session), assertiveness skills (the fourth session), stress management skills (the fifth session), positive thinking skills (the sixth session), and anger management skills (the seventh session). Educational materials were developed based on the existing literature on chronic pain management [25, 26]. Educational modules consisted of textual and audiovisual data. Each week during the study intervention, one of the CDs was provided to participants and they were asked to watch it at home in forty minutes. After watching two educational CDs, an FTF session was held for answering the questions of participants in the multimedia group. Moreover, a WhatsApp group was formed where the third author answered participants’ questions and encouraged them for using educational materials.

#### Face-to-face pain management education

For participants in the FTF group, the third author provided the same educational materials as multimedia group through the lecture, question-and-answering, and PowerPoint presentation methods in seven weekly sessions, each lasted 60–90 minutes. Participants in this group received educations in the form of small 8–10-person groups.

#### Wait-list control group

Participants in the control group received routine care services. Participants in this group were asked not to participate in any other educational programs during the study and were provided with the multimedia educational CDs at the end of the study. All participants in all groups completed the study instruments before, immediately after, and one month after the study intervention.

### Outcome measures

Data collection instruments were a demographic and clinical characteristics questionnaire, a numerical rating scale for pain intensity, and the Pain Catastrophizing Scale. The items of the demographic and clinical characteristics questionnaire were on age, gender, marital status, educational level, occupation, duration of LBP, and type of LBP treatments.

#### Primary outcome: Pain intensity

The numerical rating scale (NRS) was used to assess the average intensity of pain during the last 2 weeks. It is an eleven-point scale with 0 (“No pain”) on the one end and 10 (“Worst possible pain”) on the other. Respondents are asked to circle a point in the 0–10 range to show their pain intensity. This scale was developed by Jensen et al. and has been used in different studies for assessing different types of pain among different populations [27]. Minimal detectable change (MDC) for this scale in participants with specific back pain was calculated as 1.77 by Hasse & Kladny [28].

#### Secondary outcome: Pain catastrophizing

Previous studies showed that participants with improvement in pain intensity also experienced improvement in pain catastrophizing [29]. Therefore, pain catastrophizing was considered as the secondary outcome in the present study and was assessed using the Pain Catastrophizing Scale. The PCS was developed in 1995 by Sullivan et al. and contains thirteen items on the frequency of pain-related thoughts in three main dimensions, namely rumination (4 items), magnification (3 items), and helplessness (6 items). Items are scored on a five-point scale from 0 (“Never”) to 4 (“Always”). PCS total scores range from 0–52. The higher scores show that the client experiences more pain catastrophizing [30]. A former study reported that the Cronbach’s alpha values of the scale and its rumination, magnification, and helplessness dimensions were 0.94, 0.98, 0.78, and 0.78, respectively [31]. A study in Iran also reported that the Cronbach’s alpha of the questionnaire was 0.86 and the coefficient of correlation between the scores of this questionnaire and the scores of the short form of the Beck Depression Inventory was 0.46 [32]. The MDC for this scale was adopted from Sharma et al. (2018) study which was 6.98 [33].

### Ethical consideration

The Ethics Committee of Shiraz University of Medical Sciences, Shiraz, Iran, approved this study (code: IR-SUMS.REC.1396.117). All methods were carried out in accordance with the Declaration of Helsinki and relevant guidelines and regulations. All participants were informed about the study aim and were ensured that participation in and withdrawal from the study would be voluntary. Written informed consent was obtained from all of them. Study instruments were anonymous and were labeled with numerical codes. At the end of the study, educational CDs were provided to participants in the control group and their questions were answered.

### Statistical analysis

Data were entered into the SPSS software (v. 22.0) and analyzed at a significance level of less than 0.05. The Kolmogorov-Smirnov test was performed to test the normality of the study data. Data were described via the measures of descriptive statistics, namely mean, standard deviation, absolute frequency, and relative frequency. Groups were compared with each other using the Chi-square test (for categorical variables) or the one-way analysis of variance (for numerical variables). To analyze treatment effects, a repeated-measures multivariate analysis of variance (MANOVA) with Tukey’s post hoc test was performed. Effect sizes were reported where appropriate and calculated by Partial ɳ^2^. The following are small, medium, and large effects for ɳ^2^, respectively: .01, .06, and .14 [34]. Clinical outcome (MDC) for pain intensity and catastrophizing was also reported. MDC means that the participants with a change score smaller or equal to the MDC have a chance of more than 95% that no real change has occurred.

The regression model used in the present study was Generalized Estimating Equations (GEE). GEE procedure extends the generalized linear model to allow for analysis of repeated measurements or other correlated observations. To do so, after choosing the “Generalized estimating equations”, in the “*Repeated”* tab, the time was entered to the within-subject variables box, in the “*Type of mode”*, the linear icon was chosen in the scale response box, and in the “*Response”* tab, the pain intensity/pain catastrophizing was entered in the dependent menu. In the “*Predictors”* menu, we entered the predictors “group, pain catastrophy and time” for pain intensity and “group, pain intensity and time” as the predictors for pain catastrophy.

## Findings

**Fig 1:**
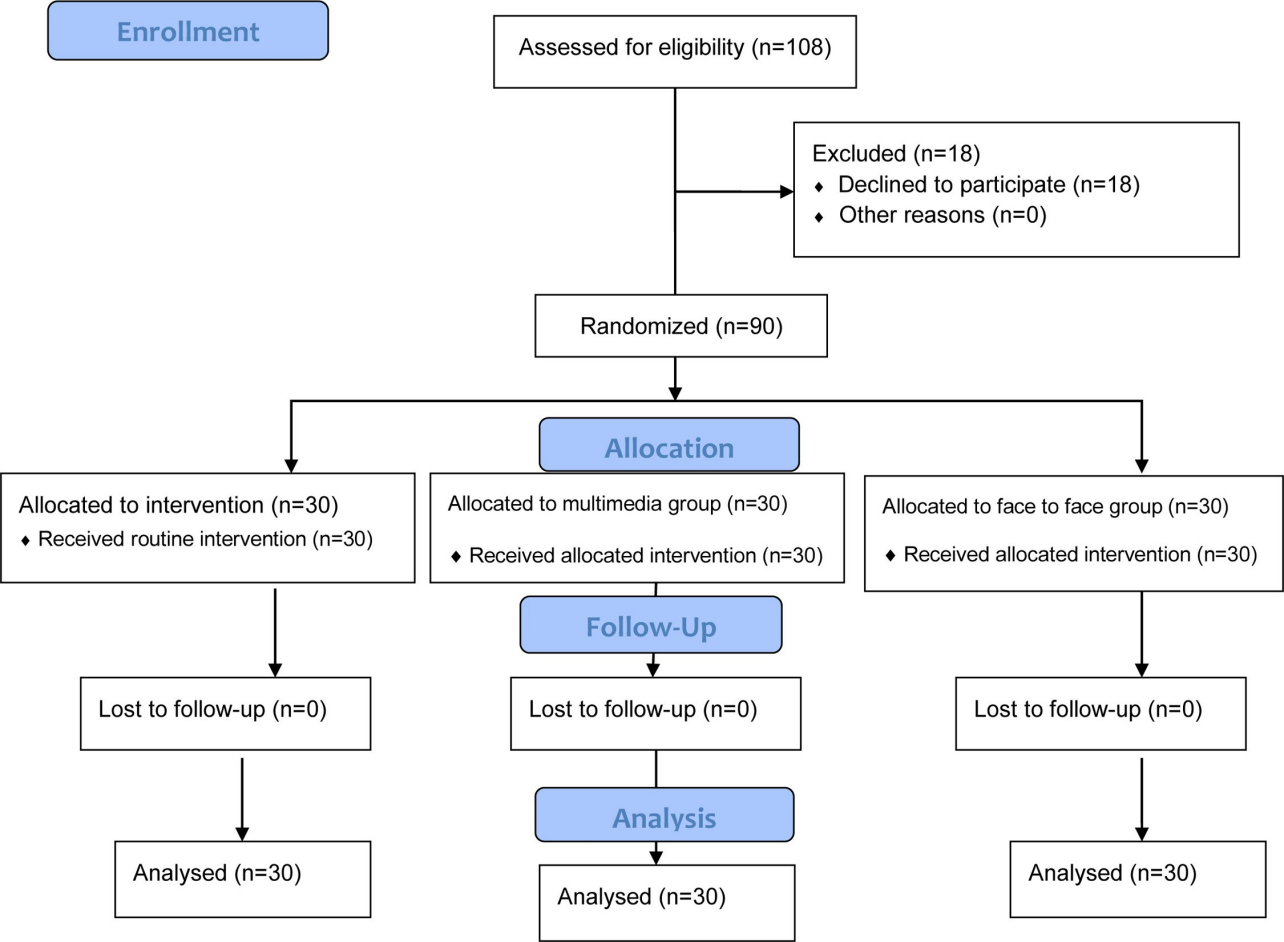
CONSORT flow diagram.

Initially, 36 participants with chronic non-specific LBP were recruited to each study group. Six participants voluntarily withdrew from each study group before the onset of the study intervention and hence, thirty participants in each group completed the study (Fig 1). The mean of participants’ age was 50.52±10.3 years and most of them were female (76.7%) and married (71.1%). Around two fifth of participants held bachelor’s degree or higher (41.1%) and were employee (43.3%). Study groups did not significantly differ from each other respecting participants’ demographic characteristics (P > 0.05; Table 1). The Kolmogorov-Smirnov test showed the normal distribution of the numerical variables.

**Table 1:** Comparison of the study groups respecting participants’ demographic characteristics and LBP duration.

Group Characteristics		FTF (n = 30)	Multimedia (n = 30)	Control (n = 30)	Total (n = 90)	Test results
Age (Years)		47.4±7.5	51±9.7	53.2±12.6	50.5±10.3	F = 2.5
Mean±SD						P = 0.09
Gender N (%)	Male	24 (26.7)	25 (27.8)	20 (22.2)	69 (76.7)	χ2 = 2.6
	Female	6(6.7)	5(5.6)	10(11.1)	21(33.3)	P = 0.27
Marital status N (%)	Single	10(11.1)	4(4.4)	2(2.2)	16(17.8)	Z = 11.14 P = 0.06
	Married	19(21.1)	20(22.2)	25(27.8)	64(71.1)	
	Widowed or divorced	1(1.1)	6(6.7)	3(3.3)	10(11.1)	
Educational level N (%)	Below diploma	7(7.8)	7(7.8)	5(5.6)	19(21.1)	Z = 5.5 P = 0.73
	Diploma	4(4.4)	11(12.2)	10(11.1)	25(27.8)	
	Associate degree	3(3.3)	2(2.2)	4(4.4)	9(10)	
	Bachelor’s degree and higher	16(17.8)	10(11.1)	11(12.2)	37(41.1)	
	Employee	17(18.9)	10(11.1)	12(13.3)	39(43.3)	
	Housewife	9(10)	12(13.3)	11(12.2)	32(35.6)	
	Retired	2(2.2)	4(4.4)	5(5.6)	11(12.2)	
	Self-employed or worker	2(2.2)	4(4.4)	2(2.2)	8(8.9)	
LBP duration		7.57±6.5	8.23±6	8.6±5.2	8.13±5.9±	F = 0.23
Mean±SD						P = 0.79

The results of MANOVA indicated that there were significant main effects of time in 3 groups (Wilks’ Lambda = 0.754, P<0.001, Partial ɳ^2^ = 0.132) on ratings of pain intensity (Table 2). This means that ignoring the effect of time, there were significant differences among the groups considering marginal means of pain intensity. Tukey’s post-hoc tests showed that there were significant differences between the control group with the FTF (p<0.001) and the multimedia group (p = 0.001) at post-treatment, but not at follow-up (p>0.05). Also, the results revealed no significant differences between the multimedia and the FTF groups at post-treatment (p = 0.994) and follow-up (p = 0.646) (Table 3). While 40.3% of the participants in all groups showed improvement in pain intensity from pretest to immediately after and 22% from immediately after to one month after the intervention, the clinical outcome measured by MDC showed that mean differences for FTF, multimedia and control group were not above the cut-off point (1.77).

**Table 2:** Comparison of the study variables in the three groups (MANOVA).

Group Variable	FTF (n = 30)			Multimedia (n = 30)			Control (n = 30)			P-value	Partial ɳ^2^	observed power
	Mean (SD)			Mean (SD)			Mean (SD)					
	Before	Immediately After	One month after	Before	Immediately After	One month after	Before	Immediately After	One month after			
Pain intensity		5.43 (1.73)	4.33 (1.56)	3.9 (1.29)	5.43 (2.16)	4.37 (1.93)	4.1 (1.64)	4.9 (2.18)	5.13 (1.96)	4.97 (1.93)	<0.001	0.116	0.977
Mean difference (SD)	Pre/immediately after	1.1 (1.35)			1.066 (1.08)			-0.23 (1.406)			<0.001	0.194	0.986
	Immediately/one month after	0.43 (0.57)			0.266 (0.827)			0.166 (0.746)			0.357	0.023	0.227
pain catastrophizing		25.6 (10.48)	17 (8.94)	16.1 (8.86)	22.87 (13.5)	16.4 (10.46)	15.73 (10.6)	19.13 (10.47)	20.9 (10.32)	20.06 (9.9)	<0.001	0.122	0.983
Mean difference(SD)	Pre/immediately after	8.6 (8.51)			6.46 (9.28)			-1.766 (8.73)			<0.001	0.209	0.992
	Immediately/one month after	0.9 (3.3)			0.67 (4.79)			0.83 (3.18)			0.971	0.001	0.054

**Table 3:** The results of Tukey’s test.

Dependent Variable		group	group	Mean Difference	P value	95% Confidence Interval	
						Lower Bound	Upper Bound
pain intensity	Pre/immediately after	FTF	multimedia	0.033	0.994	-0.758	0.82
			control	1.33	<0.001	0.54	2.12
		Multimedia	FTF	-0.33	0.994	-0.825	0.758
			control	1.3	0.001	0.508	2.09
	Post/one month after	FTF	multimedia	0.167	0.646	-0.278	0.61
			control	0.267	0.33	-0.18	0.71
		Multimedia	FTF	-0.167	0.646	-0.278	0.611
			control	0.1	0.854	0.345	0.545
pain catastrophizing	Pre/immediately after	FTF	Multimedia	2.133	0.62	-3.31	7.58
			control	10.36	<0.001	4.92	15.81
		Multimedia	FTF	-2.13	0.62	-7.58	3.31
			control	8.23	0.001	2.79	13.68
	Post/one month after	FTF	multimedia	0.233	0.97	-2.12	2.59
			control	0.0667	0.998	-2.29	2.42
		Multimedia	FTF	-0.233	0.97	-2.59	2.12
			control	-0.167	0.984	-2.52	2.19

The results of MANOVA also indicated there were significant main effects of time in 3 groups (Wilks’ Lambda = 0.771, P<0.001, Partial ɳ^2^ = 0.122) on ratings of pain catastrophizing (Table 2), suggesting that there were significant differences among the groups considering marginal means of pain catastrophizing. Tukey’s post-hoc tests showed that there were significant differences between the control group with the FTF (p<0.001) and the multimedia group (p = 0.001) at post-treatment, but not at follow-up (p>0.05). Also, the results revealed no significant differences between the multimedia and the FTF groups at post-treatment (p = 0.62) and follow-up (p = 0.97). Regarding the clinical outcome, 61% of all participants showed changes in pain catastrophizing from pretest to immediately after intervention and 35.3% from immediately to one month after intervention. Meanwhile, the mean differences changes between pretest and immediately after intervention in the FTF group was above the 6.98 points (8.6±8.51) showing the clinical improvement; however, other groups didn’t show such clinical differences.

The findings of GEE indicated that the pain intensity and catastrophizing significantly reduced across the time and between three groups. It also depicted that time and pain catastrophizing were significantly affected the pain intensity (p<0.001); however, the group was not a significant factor (p = 0.559). Furthermore, time and pain intensity significantly affected pain catastrophizing (p = 0.02 and p<0.001, respectively); meanwhile, group was not a significant factor (p = 0.81) (Table 4).

**Table 4:** Effect of intervention and covariate factors on pain catastrophizing and pain intensity using generalized estimating equations (GEE).

Variable	B	SE	X^2^	p
***Pain intensity***				
Group				
FTF	-0.774	0.4198	3.399	0.065
Multimedia	-0.57	0.4639	1.511	0.219
Control	redundant			
Time				
pretest	-0.51	0.2839	0.033	0.856
immediately after intervention	0.11	0.1317	0.693	0.405
one month after intervention	redundant			
Pain catastrophizing	0.068	0.0156	19.312	<0.001
***Pain catastrophizing***				
Group				
FTF	-0.781	2.3026	0.115	0.734
Multimedia	-2.509	2.49	1.011	0.315
Control	redundant			
Time				
pretest	-0.126	1.559	0.007	0.936
immediately after intervention	0.482	0.599	0.649	0.421
one month after intervention	redundant			
Pain intensity	2.105	0.601	12.274	<0.001

## Discussion

This study showed that both multimedia and FTF PME significantly reduced pain intensity and catastrophizing among the patients with non-specific chronic LBP, while both pain intensity and catastrophizing significantly increased in the control group, which were measured before, immediately after, and one month after the intervention. This statistically significant positive effect was confirmed by the clinically significant improvement in pain catastrophizing in the FTF PME group. These findings are in line with the findings of previous studies. For instance, a study reported the effectiveness of web-based self-management education in reducing pain intensity and catastrophizing among patients with chronic LBP [35].

The positive effects of both FTF and multimedia PME methods in the present study are attributable to not only the educational methods, but also the educational materials. The existing literature also approves that FTF education provides instructors and learners with the opportunity to verbally and non-verbally share their ideas [36]. Besides, multidimensional interventions can be more effective than uni-dimensional interventions which focus on the physical aspects of disorders [37]. The PME in the present study was also multidisciplinary because it included education about assertiveness, stress management, positive thinking, and effective communication, therefore, it can be claimed that a multidisciplinary approach was acknowledged.

Assertiveness is a key factor in interpersonal communication and anger management and hence, assertiveness education is considered as a strategy with potential positive effects on stress and anger [38]. Adaptive assertiveness helps patients with chronic pain accept their pain and prevents their pain-related abrupt and abnormal behaviors. On the other hand, adaptive assertiveness is a method to strengthen interpersonal communications because assertive individuals have greater enjoyment in their social relationships. Assertiveness also helps individuals better evaluate themselves and therefore, assertiveness education is among the techniques for reducing emotional stress [39].

Moreover, studies show that positive personality characteristics like optimism and self-confidence can reduce the negative effects of chronic pain [40]. High quality interpersonal relationships, open interactions, and symptom validation can also positively affect pain rehabilitation [41]. In fact, the continuity of important relationships in life can reduce the negative effects of pain on individuals’ sense of self. Positive thinking also helps individuals positively interpret their pain and thereby, reduces the intensity and catastrophizing of pain among patients with chronic back pain [40]. Anger is also known as an inseparable part of the pain experience and is very common among patients with chronic pain. High trait anger-out is in turn associated with greater pain responsiveness [42]. Therefore, psychological techniques such as thoughtful challenging exposure to difficult tasks, mindfulness, and acceptance can be used to improve mood status and facilitate the use of other pain management skills among patients with pain [43].

There are limited studies into the effects of multimedia PME on pain catastrophizing among patients with chronic LBP. A previous study conducted using internet-based education and telephone support reported positive effect in reducing pain catastrophizing among patients with chronic back pain, though researchers reported their inability to have continuous contact with patients throughout the study [44]. Contrarily, we made frequent telephone contacts with participants in order to encourage them for using educational materials and answer their questions.

Despite little evidence regarding the impact of PME on pain catastrophizing, there is ample documents that pain catastrophizing plays an important role in the experience of pain. For example, studies have shown that pain catastrophizing is a strong predictor of pain disability, hospital discharge time, postoperative pain intensity, and quality of life [45]. Therefore, evaluating other consequences e.g. quality of life and disability caused by chronic pain along with pain catastrophizing, especially in longitudinal studies, should be warranted in the future studies.

Study findings also revealed that although the mean scores of pain intensity and catastrophizing significantly decreased in both intervention groups, there was no statistically significant difference between these groups. Nonetheless, the time-saving attribute of online methods, such as the multimedia method, helps healthcare providers allocate their time to their other professional activities rather than FTF education [46].

Of course, the findings of the present study should be interpreted based on the measurement reliability of the study. Measurement reliability is an influential factor on sample size, effect size, and study power [47]. The reliability of the data collection instruments in the present study was confirmed with acceptable test-retest and inter-rater correlation coefficients (i.e., more than 0.8) [48]. Therefore, sample size had been calculated with acceptable measurement reliability. Nonetheless, future studies are recommended to use different measurement instruments to improve measurement reliability.

Furthermore, as chronic pain is associated with different psychological consequences, PME should be provided over a long period of time and patients should be monitored for longer periods of time, for example more than three months. Therefore, studies with long-term interventions are needed to produce more scientific evidence regarding the effects of PME on chronic pain.

A strength of the present study was that as a clinical trial, it provides reliable data about causal relationships between pain management training and pain. Another strength of this study was that it reduced the gap related to the paucity of data about the effects of PME on LBP in Asian and Iranian contexts. However, this study faced some limitations. One of the limitations of the present study was that participants’ disability and physical functionality were not assessed, while the biopsychosocial model of pain holds that chronic pain has physical, psychological, emotional, and social aspects and effects. Pain intensity and pain catastrophizing were assessed in the present study as two aspects of pain. Functionality, sleep, and mobility can be assessed in future studies using The Brief Pain Inventory and the West Haven Yale Multidimensional Pain Inventory. Another limitation of the study was the use of self-report instruments for data collection. Some patients may overestimate of underestimate their pain intensity during self-report assessment. Moreover, some effects of PME on chronic pain may appear after a long of time, while we monitored patients only for one month. Assessment of the long-term clinical effects of PME on other pain outcomeslike physical activity can provide better understanding of the effects of pain on daily life and help develop more effective pain management strategies.

Furthermore, although some earlier studies considered a cutoff point for the score of pain catastrophizing, we analyzed pain catastrophizing-related data without considering any cutoff point and hence, comparison of the study findings with the findings of other studies may be challenging or difficult. The other limitation of the study was that it was performed on a relatively small sample size; hence, this sample probably could not be representative of patients with LBP in different cities in Iran.

## Conclusion

This study showed that both multimedia and FTF PME have potential in relieving pain intensity and catastrophizing among Iranian participants with chronic LBP. Given the high prevalence of chronic LBP in different populations, using multimedia and FTF methods is recommended for providing afflicted participants with PME about chronic LBP. In pain management programs, special attention should be paid not only to the physical aspects of pain, but also to its psychological aspects. In other words, a comprehensive holistic approach should be used in pain clinics for more effective pain management. Studies on larger and more diverse samples of participants can provide more reliable information about the effects of PME on pain.

Future studies into chronic LBP management are recommended to assess the effects of PME on different aspects of pain such as pain-related interference, pain tolerance, analgesic use, and quality of life. Moreover, studies with similar pain intensity and catastrophizing assessment methods are recommended to produce comparable results.

## Supporting information

S1 Checklist CONSORT checklist. (DOC)

S1 File Trial protocol. (DOCX)
