# Supplementary material for: Comparing the effects of multimedia and face-to-face pain management education on pain intensity and pain catastrophizing among patients with chronic low back pain: A randomized clinical trial
Source: PLoS One. 2022 Jun 16;17(6):e0269785. doi: 10.1371/journal.pone.0269785 (PMC9202919; doi:10.1371/journal.pone.0269785)
Supplement: S1 File — (DOCX) [file pone.0269785.s002.docx]

Research protocol

**Project summary**

**Background:** Low back pain (LBP) is a very common problem among different age groups. Previous studies into LBP assessed the effects of physical interventions or face-to-face (FTF) education mostly in western cultures.

**Study aim**

The present study aim to compare to compare the effects of multimedia and FTF pain management education (PME) on pain intensity and pain catastrophizing among patients with chronic LBP.

**Design**Double-blind randomized controlled clinical trial on ninety patients with chronic LBP. Participants will be randomly allocated to either multimedia, FTF, or control groups through block randomization.

**Settings and conduct**

Participants in the multimedia group will receive PME through watching seven educational CDs at home and their counterparts in the FTF group receive the same educations in seven weekly FTF educational sessions. Pain intensity and pain catastrophizing will be assessed before, immediately after, and one month after the study intervention.

**General information**

Protocol title: **Comparing the effects of multimedia and face-to-face pain management education on pain intensity and pain catastrophizing among patients with chronic low back pain: a randomized clinical trial**

Name and address of the sponsor/funder: Shiraz University of Medical Sciences

Address: Vice-Chancellor for Research, Shiraz University of Medical Sciences, Zand Blvd, Shiraz, Iran. Postal code: 7134814336

Name and title of the investigator(s):

Maryam Shaygan^a^, Azita Jaberi^b,*^, Roghayyeh Firozian ^c^, Zahra Yazdani^d^

^a^ Community Based Psychiatric Care Research Center, Shiraz University of Medical Sciences, Shiraz, Iran

E-mail: [m2620.shaygan@gmail.com](mailto:m2620.shaygan@gmail.com)

^b,*^ (BS.c, MS.c, PhD), Community Based Psychiatric Care Research Center, Shiraz University of Medical Sciences, Shiraz, Iran

Phone: +98(713)6474255-8
E-mail: [azita635@yahoo.com](mailto:azita635@yahoo.com)

^c^ Student Research Committee, Shiraz University of medical sciences, Shiraz, Iran

Phone +987136474256, E-mail: [firoozianelahe@gmail.com](mailto:firoozianelahe@gmail.com)

^d^ Department of Nursing, School of Nursing and Midwifery, Shiraz University of Medical Sciences, Shiraz, Iran

**Rationale & background information**

Low back pain (LBP) is a common type of chronic pain ([Zernikow, Kriszio, Frosch, Dobe, & Wager, 2019](#_ENREF_32)) which affects 57.6 million people in the world ([Vos et al., 2017](#_ENREF_30)). LBP imposes a heavy burden on healthcare systems so that almost three fourth of all pain-related healthcare costs are spent on LBP management ([Fourney et al., 2011](#_ENREF_9)). It not only causes physical and financial problems, but also negatively affects the mental status and the social activities of afflicted patients and their families ([Ojeda et al., 2014](#_ENREF_19)).

We know that chronic pain has different biopsychosocial aspects and hence, its management should be based not only on physical factors, but also on psycho-social factors ([Ramond et al., 2011](#_ENREF_20)). The Fear Avoidance Model illustrates that some psychological factors such as pain catastrophizing can lead to avoidance, depression, and disability among patients with chronic pain ([Linton & Shaw, 2011](#_ENREF_16)).

Pain catastrophizing is a cognitive process of exaggerating or magnifying pain perception which results in greater attention to the negative aspects of pain, interpretation of physical arousals as pain symptoms, and pain intensity ([Turner & Aaron, 2001](#_ENREF_29)). Besides pain catastrophizing, factors such as stress, anger, poor communication skills, and limited assertiveness can affect pain intensity among patients with chronic conditions. For example, a study found stress, social conflicts, and non-assertive relationships as aggravating factors of chronic pain ([Ashton-James & Ziadni, 2020](#_ENREF_2)). Studies into occupational therapy for chronic pain also emphasize that patients with limited problem solving, communication, and assertiveness skills may have problems in pain management ([Hill, 2016](#_ENREF_12)). Consequently, besides physical interventions, education of psychological skills such as stress management, assertiveness, effective communication, positive thinking, and anger management should be included in pain management programs ([Andersson, Johansson, Nordlander, & Asmundson, 2012](#_ENREF_1)).

Previous studies reported contradictory results respecting the effects of outpatient psychological interventions such as cognitive behavioral therapy on chronic pain management in adults ([Tse, Yeung, Lee, & Ng, 2016](#_ENREF_28)). For example, a systematic review on nineteen studies found that there was no clear evidence concerning the effects of cognitive behavioral therapy, biofeedback, and relaxation on pain intensity and disability among patients with neck pain ([Shearer et al., 2016](#_ENREF_23)). A recent meta-analysis on eleven clinical trials also found no significant difference between the long-term effects of psychological interventions and standard interventions on pain intensity ([Bérubé et al., 2021](#_ENREF_3)). Besides contradictory results, previous studies mostly focused on one aspect of pain (either physical or psychological), while chronic pain is a complex experience and hence multidisciplinary interventions are needed for its effective management ([Zernikow et al., 2019](#_ENREF_32)). On the other hand, patients who live far from pain management centers may have limited access to multidisciplinary pain management services. Therefore, tele-nursing methods such as multimedia education are needed to improve patient access to pain management services and remove the barriers to evidence-based treatments.

Some previous studies evaluated the effects of technology-based pain management programs ([Garg, Garg, Turin, & Chowdhury, 2016](#_ENREF_10); [Vugts, Joosen, van der Geer, Zedlitz, & Vrijhoef, 2018](#_ENREF_31)). However, most of them were web-based programs ([Garg et al., 2016](#_ENREF_10); [Nevedal, Wang, Oberleitner, Schwartz, & Williams, 2013](#_ENREF_18)), single-center, and non-controlled ([Bérubé et al., 2021](#_ENREF_3); [Shearer et al., 2016](#_ENREF_23)). Moreover, there are limited comparative studies into the effects of FTF and multimedia pain management programs ([Suman, Schaafsma, Bamarni, Van Tulder, & Anema, 2017](#_ENREF_26); [Suman et al., 2018](#_ENREF_27)). On the other hand, the results of the existing studies in this area may not be generalizable to different context because the effects of psychological factors on chronic pain largely depend on patients’ sociocultural background ([Kovacs et al., 2012](#_ENREF_15)). Nonetheless, most previous studies into pain catastrophizing and pain were conducted in northern European countries and Anglo-Saxon cultures ([Hirsh, George, Riley III, & Robinson, 2007](#_ENREF_13); [Moix, Kovacs, Martín, Plana, & Royuela, 2011](#_ENREF_17)). There is limited information in this area in the culture of Asian countries, particularly in Iran. Therefore, studies in different contexts and cultures are needed to provide more reliable results regarding the effects of multimedia pain management program on chronic LBP and pain catastrophizing ([Kovacs et al., 2012](#_ENREF_15)). All these gaps highlight the necessity of conducting further studies into pain catastrophizing among patients with chronic LBP and the comparative effects of face-to-face and multimedia treatments in this area. The present study was designed and conducted to reduce these gaps. The aim of the current study was to compare the effects of multimedia and face to face (FTF) pain management education (PME) on pain intensity and pain catastrophizing among patients with chronic LBP. The hypothesis of the present study was that patients with chronic back pain who receive FTF and multimedia PME will show significant decrease in pain intensity and pain catastrophizing, assessed immediately after program and at 1-month follow-up.

**References (of literature cited in preceding sections)**

Andersson, Gerhard, Johansson, Carina, Nordlander, Annette, & Asmundson, Gordon JG. (2012). Chronic pain in older adults: A controlled pilot trial of a brief cognitive-behavioural group treatment. *Behavioural and cognitive psychotherapy, 40*(2), 239-244.

Ashton-James, Claire E, & Ziadni, Maisa S. (2020). Uncovering and Resolving Social Conflicts Contributing to Chronic Pain: Emotional Awareness and Expression Therapy. *Journal of Health Service Psychology, 46*(3), 133-140.

Bérubé, Mélanie, Martorella, Géraldine, Côté, Caroline, Gélinas, Céline, Feeley, Nancy, Choinière, Manon, . . . Streiner, David L. (2021). The Effect of Psychological Interventions on the Prevention of Chronic Pain in Adults: A Systematic Review and Meta-analysis. *The Clinical Journal of Pain, 37*(5), 379-395.

Chibnall, John T, & Tait, Raymond C. (2005). Confirmatory factor analysis of the Pain Catastrophizing Scale in African American and Caucasian Workers' Compensation claimants with low back injuries. *Pain, 113*(3), 369-375.

Clark, Laura, Schmidt, Ulrike, Tharmanathan, Puvan, Adamson, Joy, Hewitt, Catherine, & Torgerson, David. (2013). Poor reporting quality of key Randomization and Allocation Concealment details is still prevalent among published RCTs in 2011: a review. *Journal of evaluation in clinical practice, 19*(4), 703-707.

Davoudi, I, Zargar, Y, Mozaffaripour, E, Nargesi, F, & Molah, K. (2012). The Relationship between pain catastrophizing, social support, pain-related anxiety, coping strategies and neuroticism, with functional disability in rheumatic patients.

Dobson, K.S. (2010). *Handbook of Cognitive-Behavioral Therapies*. New York: The Guilford express.

Dobson, K.S., & Dozois, D.J.A. (2010). *Historical and philosophical bases of the cognitive-behavioral therapies*: Guilford Press.

Fourney, Daryl R, Andersson, Gunnar, Arnold, Paul M, Dettori, Joseph, Cahana, Alex, Fehlings, Michael G, . . . Chapman, Jens R. (2011). Chronic low back pain: a heterogeneous condition with challenges for an evidence-based approach. *Spine, 36*, S1-S9.

Garg, Shashank, Garg, Divya, Turin, Tanvir C, & Chowdhury, M Faruq U. (2016). Web-based interventions for chronic back pain: a systematic review. *Journal of medical Internet research, 18*(7), e139. doi: 10.2196/jmir.4932

Haase, Ingo, & Kladny, Bernd. (2021). Clinical relevance of changes in pain intensity in patients with specific back pain. *Zeitschrift für Orthopädie und Unfallchirurgie*.

Hill, Wendy. (2016). The role of occupational therapy in pain management. *Anaesthesia & Intensive Care Medicine, 17*(9), 451-453.

Hirsh, Adam T, George, Steven Z, Riley III, Joseph L, & Robinson, Michael E. (2007). An evaluation of the measurement of pain catastrophizing by the coping strategies questionnaire. *European Journal of Pain, 11*(1), 75-81.

Jensen, M.P., Karoly, P., & Braver, S. (1986). The measurement of clinical pain intensity: a comparison of six methods. *Pain, 27*(1), 117-126. doi: 10.1016/0304-3959(86)90228-9

Kovacs, Francisco M, Seco, Jesús, Royuela, Ana, Corcoll-Reixach, Josep, Peña-Arrebola, Andrés, & Network, Spanish Back Pain Research. (2012). The prognostic value of catastrophizing for predicting the clinical evolution of low back pain patients: a study in routine clinical practice within the Spanish National Health Service. *The Spine Journal, 12*(7), 545-555.

Linton, Steven J, & Shaw, William S. (2011). Impact of psychological factors in the experience of pain. *Physical therapy, 91*(5), 700-711.

Moix, Jenny, Kovacs, Francisco M, Martín, Andrés, Plana, María N, & Royuela, Ana. (2011). Catastrophizing, state anxiety, anger, and depressive symptoms do not correlate with disability when variations of trait anxiety are taken into account. a study of chronic low back pain patients treated in Spanish pain units [NCT00360802]. *Pain Medicine, 12*(7), 1008-1017.

Nevedal, Dana C, Wang, Chun, Oberleitner, Lindsay, Schwartz, Steven, & Williams, Amy M. (2013). Effects of an individually tailored web-based chronic pain management program on pain severity, psychological health, and functioning. *Journal of medical Internet research, 15*(9), e201. doi: 10.2196/jmir.2296

Ojeda, Begoña, Salazar, Alejandro, Dueñas, María, Torres, Luís Miguel, Micó, Juan Antonio, & Failde, Inmaculada. (2014). The impact of chronic pain: The perspective of patients, relatives, and caregivers. *Families, Systems, & Health, 32*(4), 399.

Ramond, Aline, Bouton, Celine, Richard, Isabelle, Roquelaure, Yves, Baufreton, Christophe, Legrand, Erick, & Huez, Jean-François. (2011). Psychosocial risk factors for chronic low back pain in primary care—a systematic review. *Family practice, 28*(1), 12-21.

Sharma, Saurab, Thibault, Pascal, Abbott, J Haxby, & Jensen, Mark P. (2018). Clinimetric properties of the Nepali version of the Pain Catastrophizing Scale in individuals with chronic pain. *Journal of pain research, 11*, 265.

Shaygan, Maryam, Böger, Andreas, & Kröner-Herwig, Birgit. (2019). How does reduction in pain lead to reduction in disability in patients with musculoskeletal pain? *Journal of pain research, 12*, 1879.

Shearer, Heather M, Carroll, Linda J, Wong, Jessica J, Cote, Pierre, Varatharajan, Sharanya, Southerst, Danielle, . . . Mior, Silvano A. (2016). Are psychological interventions effective for the management of neck pain and whiplash-associated disorders? A systematic review by the Ontario Protocol for Traffic Injury Management (OPTIMa) Collaboration. *The Spine Journal, 16*(12), 1566-1581.

Stevens, J. (1996). *Applied multivariate statistics for the social sciences.* NJ: Erlbaum: Mahwah.

Sullivan, Michael JL, Bishop, Scott R, & Pivik, Jayne. (1995). The pain catastrophizing scale: development and validation. *Psychological assessment, 7*(4), 524.

Suman, Arnela, Schaafsma, Frederieke G, Bamarni, Jiman, Van Tulder, Maurits W, & Anema, Johannes R. (2017). A multimedia campaign to improve back beliefs in patients with non-specific low back pain: a process evaluation. *BMC Musculoskeletal Disorders, 18*(1), 1-13.

Suman, Arnela, Schaafsma, Frederieke G, Van De Ven, Peter M, Slottje, Pauline, Buchbinder, Rachelle, Van Tulder, Maurits W, & Anema, Johannes R. (2018). Effectiveness of a multifaceted implementation strategy compared to usual care on low back pain guideline adherence among general practitioners. *BMC health services research, 18*(1), 1-9.

Tse, Mimi Mun Yee, Yeung, Suey Shuk Yu, Lee, Paul Hong, & Ng, Shamay Sheung Mei. (2016). Effects of a peer-led pain management program for nursing home residents with chronic pain: A pilot study. *Pain Medicine, 17*(9), 1648-1657.

Turner, Judith A, & Aaron, Leslie A. (2001). Pain-related catastrophizing: what is it? *The Clinical journal of pain, 17*(1), 65-71.

Vos, Theo, Abajobir, Amanuel Alemu, Abate, Kalkidan Hassen, Abbafati, Cristiana, Abbas, Kaja M, Abd-Allah, Foad, . . . Abera, Semaw Ferede. (2017). Global, regional, and national incidence, prevalence, and years lived with disability for 328 diseases and injuries for 195 countries, 1990–2016: a systematic analysis for the Global Burden of Disease Study 2016. *The Lancet, 390*(10100), 1211-1259.

Vugts, Miel AP, Joosen, Margot CW, van der Geer, Jessica E, Zedlitz, Aglaia MEE, & Vrijhoef, Hubertus JM. (2018). The effectiveness of various computer-based interventions for patients with chronic pain or functional somatic syndromes: A systematic review and meta-analysis. *PloS one, 13*(5), e0196467.

Zernikow, Boris, Kriszio, Holger, Frosch, Michael, Dobe, Michael, & Wager, Julia. (2019). Pain Disorder: A Biopsychosocial Disease *Practical Treatment Options for Chronic Pain in Children and Adolescents* (pp. 7-34): Springer.

**Study goals and objectives**

**Goal:**

Comparing the effects of multimedia and FTF pain management education (PME) on pain intensity and pain catastrophizing (PC) among patients with chronic LBP.

**Objectives:**

- Determining the demographic variables of participants
- Determining and comparing the mean score of pain intensity within the interventions and control groups, before, immediately and one month after intervention
- Comparing the changes of the mean score of pain intensity between the intervention and control groups
- Determining and comparing the mean score of pain catastrophizing within the interventions and control groups, before, immediately and one month and after intervention
- Comparing the changes of the mean score of pain catastrophizing between the interventions and control groups

**Study design**

The participants will be randomly assigned to three groups each containing 36 participants receiving pain management education via multimedia or face to face (FTF) method and in control group no education will be performed.

**Participants**

The participants of the current study will be males and females aged over 18 years with non-specific chronic LBP recruited from three healthcare centers. All interested patients will be invited to participate in the research. Afterwards, the interested individuals will be selected based on other inclusion and exclusion criteria.

**Inclusion criteria:**

- age over eighteen years
- agreement for participation
- ability to use educational CDs
- ability to attend the study setting for participation in FTF PME sessions
- no affliction by other types of chronic pain (such as chronic headache
- no affliction by chronic psychological disorders (such as schizophrenia)
- definite diagnosis of non-specific chronic LBP established at least one month before the study by a medical specialist based on the data obtained through physical examination and diagnostic procedures such as simple radiography, computed tomography, or magnetic resonance imaging

**Exclusion criteria:**

- voluntary withdrawal from the study for any reason (such as severe LBP)
- participation in any other educational program on pain during the study
- more than two absences from the educational sessions of the study intervention.

**Expected recruitment start date**

2018-05-10
**Expected recruitment end date**

2019-05-10

**Methodology**

**Randomization description**

Participants will be randomly allocated to either the multimedia, FTF, or the wait-list control group. A research assistant not involved in the current study will perform random allocation through block randomization with a block size of 6 and cards labeled A, B, and C. Allocation concealment will be ensured using sequentially numbered, opaque, sealed envelopes (SNOSE) ([Clark et al., 2013](#_ENREF_5)).
**Blinding**The patients will be blinded to the patient group assignments and will not know what the other interventions will be. In addition, the evaluator and the analyzer of the outcomes will not be informed about the patients’ treatment assignments.

**Interventions**

***Multimedia pain management education***

Eligible participants in this group will receive multimedia PME in seven weeks. The modules of this education will consist of definition of chronic pain and the psychological factors affecting pain experience (the first session), appropriate physical exercises for chronic LBP (the second session), effective communication skills (the third session), assertiveness skills (the fourth session), stress management skills (the fifth session), positive thinking skills (the sixth session), and anger management skills (the seventh session). Educational materials will be developed based on the existing literature on chronic pain management ([Dobson, 2010](#_ENREF_7); [Dobson & Dozois, 2010](#_ENREF_8)). Educational modules will consist of textual and audiovisual data. Each week during the study intervention, one of the CDs will be provided to participants and they will be asked to watch it at home in forty minutes. After watching two educational CDs, an FTF session will be held for answering the questions of participants in the multimedia group. Moreover, a WhatsApp group will be formed where the third author answered patients’ questions and encouraged them for using educational materials.

***Face-to-face pain management education***

For participants in the FTF group, the third author will provide the same educational materials as multimedia group through the lecture, question-and-answering, and PowerPoint presentation methods in seven weekly sessions, each last 60–90 minutes. Participants in this group will receive educations in the form of small 8–10-person groups.

***Wait-list control group***

Participants in the control group will receive routine care services. Participants in this group will be asked not to participate in any other educational programs during the study and will be provided with the multimedia educational CDs at the end of the study. All participants in all groups will complete the study instruments before, immediately after, and one month after the study intervention.

**Safety considerations**

All methods will be carried out in accordance with the Declaration of Helsinki and relevant guidelines and regulations. All participants will be informed about the study aim and ensured that participation in and withdrawal from the study would be voluntary. Written informed consent will be obtained from all of them. Study instruments will be anonymous and be labeled with numerical codes. At the end of the study, educational CDs will be provided to participants in the control group and their questions will be answered.

**Follow-up**

Participants in the multimedia PME group will receive PME through watching seven educational CDs at home and their counterparts in the FTF PME group receive the same educations in seven weekly FTF educational sessions.

**Data management and statistical analysis**

Data collection instruments will be a demographic and clinical characteristics questionnaire, a numerical rating scale for pain intensity, and the Pain Catastrophizing Scale. The items of the demographic and clinical characteristics questionnaire will be on age, gender, marital status, educational level, occupation, duration of LBP, and type of LBP treatments.

***Primary outcome: Pain intensity***

The numerical rating scale (NRS) will be used to assess the average intensity of pain during the last 2 weeks. It is an eleven-point scale with 0 (“No pain”) on the one end and 10 (“Worst possible pain”) on the other. Respondents are asked to circle a point in the 0–10 range to show their pain intensity. This scale was developed by Jensen et al. and has been used in different studies for assessing different types of pain among different populations ([Jensen, Karoly, & Braver, 1986](#_ENREF_14)). Minimal detectable change (MDC) for this scale in patients with specific back pain was calculated as 1.77 by Hasse & Kladny ([Haase & Kladny, 2021](#_ENREF_11)).

***Secondary outcome: Pain catastrophizing***

Previous studies showed that patients with improvement in pain intensity also experienced improvement in pain catastrophizing ([Shaygan, Böger, & Kröner-Herwig, 2019](#_ENREF_22)). Therefore, pain catastrophizing will be considered as the secondary outcome in the present study and assessed using the Pain Catastrophizing Scale. The PCS was developed in 1995 by Sullivan et al. and contains thirteen items on the frequency of pain-related thoughts in three main dimensions, namely rumination (4 items), magnification (3 items), and helplessness (6 items). Items are scored on a five-point scale from 0 (“Never”) to 4 (“Always”). PCS total scores range from 0-52. The higher scores show that the client experiences more pain catastrophizing ([Sullivan, Bishop, & Pivik, 1995](#_ENREF_25)). A former study reported that the Cronbach’s alpha values of the scale and its rumination, magnification, and helplessness dimensions were 0.94, 0.98, 0.78, and 0.78, respectively ([Chibnall & Tait, 2005](#_ENREF_4)). A study in Iran also reported that the Cronbach’s alpha of the questionnaire was 0.86 and the coefficient of correlation between the scores of this questionnaire and the scores of the short form of the Beck Depression Inventory was 0.46 ([Davoudi, Zargar, Mozaffaripour, Nargesi, & Molah, 2012](#_ENREF_6)). The MDC for this scale was adopted from Sharma et al. (2018) study which was 6.98 ([Sharma, Thibault, Abbott, & Jensen, 2018](#_ENREF_21)).

**Data analysis**:

Data will be entered into the SPSS software (v. 22.0) and analyzed at a significance level of less than 0.05. The Kolmogorov-Smirnov test will be performed to test the normality of the study data. Data will be described via the measures of descriptive statistics, namely mean, standard deviation, absolute frequency, and relative frequency. Groups will be compared with each other using the Chi-square test (for categorical variables) or the one-way analysis of variance (for numerical variables). To analyze treatment effects, a repeated-measures multivariate analysis of variance (MANOVA) with Tukey’s post hoc test will be performed. Effect sizes will be reported where appropriate and calculated by Partial ɳ^2^. The following are small, medium, and large effects for ɳ^2^, respectively: .01, .06, and .14 ([Stevens, 1996](#_ENREF_24)). Clinical outcome (MDC) for pain intensity and catastrophizing will be also reported. MDC means that the participants with a change score smaller or equal to the MDC have a chance of more than 95% that no real change has occurred.

The regression model used in the present study will be Generalized Estimating Equations (GEE). GEE procedure extends the generalized linear model to allow for analysis of repeated measurements or other correlated observations. To do so, after choosing the “Generalized estimating equations”, in the “*Repeated”* tab, the time will be entered to the within-subject variables box, in the “*Type of mode”*, the linear icon will be chosen in the scale response box, and in the “*Response”* tab, the pain intensity/pain catastrophizing will be entered in the dependent menu. In the “*Predictors”* menu, we will enter the predictors “group, pain catastrophy and time” for pain intensity and “group, pain intensity and time” as the predictors for pain catastrophy.

### Quality assurance

The Ethics Committee of Shiraz University of Medical Sciences, Shiraz, Iran, approved this study (code: IR-SUMS.REC.1396.117). All methods will be carried out in accordance with the Declaration of Helsinki and relevant guidelines and regulations. All participants will be informed about the study aim and will be ensured that participation in and withdrawal from the study would be voluntary. Written informed consent will be obtained from all of them. Study instruments will be anonymous and labeled with numerical codes. At the end of the study, educational CDs will be provided to participants in the control group and their questions will be answered.

**Expected outcomes of the study**

If the efficacy of multimedia and FTF pain management education in improving the pain intensity and catastrophizing of the participants confirmed, these educations can be utilized in health centers. Using Multimedia PME may be safer and more beneficial and effective than FTF PME in the current COVID-19 pandemic.

**Dissemination of results and publication policy**

The principal investigators intends to present the results at national conferences and publish in peer-reviewed medical journals. Dr. Azita Jaberi will take the lead in publication and updating data.

**Duration of the project**

**Expected recruitment start date**

2018-05-10
**Expected recruitment end date**

2019-05-10

### Problems anticipated

### The probability of 20% attrition rate, which the sample size will be increased to solve this problem.

### The probability of unwillingness of participants to follow the study.

### The difficulty of learning how to use multimedia.

### Project management

MSH and AJ designed the study, FSH and AJ will supervise and direct the study, RF will carry out the implementation, RF and FSH will process the experimental data, perform the analysis and draft the manuscript, AJ will work on the manuscript. All authors will assess the results, comment on the manuscript and approve the final manuscript.

**Ethics**

The Ethics Committee of Shiraz University of Medical Sciences, Shiraz, Iran, approved this study (code: IR-SUMS.REC.1396.117). All participants will be informed about the study aim and will be ensured that participation in and withdrawal from the study would be voluntary. Written informed consent will be obtained from all of them. Study instruments will be anonymous and labeled with numerical codes. At the end of the study, educational CDs will be provided to participants in the control group and their questions will be answered.

### Informed consent forms

A session will be held after the selection of participants for explaining the study objectives and procedures. Written informed consent forms will also be taken from all participants. A sample of informed consent in Persian is presented.

**
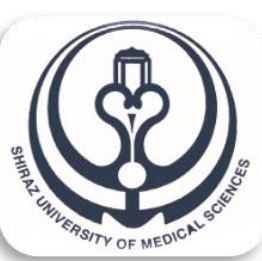
In the name of God**

Informed consent form Clinical study participation

Medical Science Research Ethics Committee of Shiraz University of Medical Sciences

| Comparing the effects of multimedia and face-to-face positive psychology on pain intensity and pain catastrophizing among patients with chronic low back pain: a randomized clinical trial | **Research title** |
| --- | --- |
| IR.SUMS.REC.1396.117 | **Research number** |
| Maryam Shaygan | **Executor(s) name(s)** |
| School of Nursing and Midwifery | **Associated faculty or unit** |
| In this research, patients with chronic LBP are given pain management education via multimedia or face to face for seven weeks. After the intervention, their pain intensity and catastrophizing will be assessed. | **Research introduction** |
| No | **Blood sampling** |
| These educations are more attractive compared to others.  Using these educations may save time and money. | **Advantages** |
| none | **Risks** |
| NA | **Risk compensation** |
| The participants will not be required to pay for the educations. | **Costs** |
| participants will receive routine cares if they do not accept to participate in these educations. | **Alternative methods** |
| While the individuals’ identities will remain confidential, the results of this research will only be utilized for research purposes, and the participants will be informed about the results.  The collected samples may be used for future research. | **Confidentiality** |
| Individuals can contact on 07136474255 in case they have any questions about the research. | **Responding to question** |
| My participation in this research is voluntary and I am free to refuse to participate or quit the research at any time without any changes in the physician’s behavior or treatment process. | **Rejection and renunciation right** |
| We sincerely thank you for participating in this research, and we would appreciate it if you let us know about any suggestions or problems in the process of conducting this research through the Ethics Committee of Shiraz University of Medical Sciences, phone number 32122686, or email address [researchethic@sums.ac.ir](mailto:researchethic@sums.ac.ir). Handling the referred problems will confidentially take place at the Ethics Committee of Shiraz University of Medical Sciences. | **Notices, suggestions, and handling problems** |
| Dear elderly/participant,  You will hereby be informed that more details about this study are available on the Iranian Clinical Trial Registration Center website, [www.irct.ir](http://www.irct.ir).  IRCT registration number: **-----** | |
| **Consent**  I .......................... declare that I will compliantly participate in this research as a study subject while having full knowledge of the above information.  All my information and my name will remain confidential, the research results will be presented as the information of the studied group, and the individual information will be provided without stating the name or personal information if needed. I declare this research's physician(s)' quittance of all the aforementioned actions in the information sheet in case there is no action negligence.  This agreement is not a means to prevent legal action against Shiraz University of Medical Sciences in case illegal or inhuman actions take place.  The studied individual's signature and finger print  Witness's full name, contact number, and signature  Researcher's full name and signature  (or the legal guardian)  Home tel. number:  Mobile phone number:  Date: | |
